# How to monitor thiopental administration in the intensive care unit for refectory status epilepticus or intracranial hypertension?

**DOI:** 10.1186/s13054-021-03851-8

**Published:** 2021-12-17

**Authors:** Erika Dabricot, Inès Seqat, Frédéric Dailler, Sylvain Rheims, Sebastien Boulogne, Baptiste Balança

**Affiliations:** 1 Department of Neurological Anesthesiology and Intensive Care, Hospices Civils de Lyon, Hôpital Pierre Wertheimer, Groupement Hospitalier Est, 59 Boulevard Pinel, 69500 Bron, Lyon, France; 2 Department of Functional Neurology and Epileptology, Hospices Civils de Lyon and Lyon 1 University, Lyon, France; 3 Lyon’s Neuroscience Research Center, INSERM U1028/CNRS UMR 5292, Lyon 1 University, Lyon, France

## Dear editor,

Thiopental continuous administration can be used as a rescue therapy for refractory status epilepticus (SE) or intracranial hypertension (IH). It induces an electroencephalographic (EEG) slowing up to a burst suppression state. The subsequent reduction in the cerebral metabolic demand and blood flow also allows decreasing the intracranial pressure (ICP) [1]. The continuous administration is usually guided both by thiopental serum concentration, to avoid accumulation, and efficacy on seizures or ICP. Thiopental side effects (hemodynamic dysfunction or immunosuppression) can occur at concentrations of 30–70 mg/ml [2, 3]. Conversely, the relation between serum concentrations and efficacy is less robust. In healthy subjects during anesthesia and in brain-injured patients, there is a great variability in the concentration needed to reach the same EEG changes [4, 5], with an overlap between the therapeutic and toxic ranges. The digitalization of the EEG signal provides quantitative indexes at the bedside and may allow tailoring sedative administration in the intensive care unit (ICU). For instance, the suppression ratio (SR) provides a metric of the depth of sedation during general anesthesia [5]. Since the target of thiopental sedation is to reach a discontinuous EEG activity (i.e., SR ≥ 10%); the aim of the herein study was to evaluate the relationship between the thiopental concentration and the SR in patients with a refractory SE or IH.

We conducted a retrospective study (2013–2020) in adult patients sedated with thiopental and monitored with a continuous EEG, in the neurological ICU of the Hospices Civils de Lyon (France). We analyzed EEG signals of 2 h windows around the serum measurements (1 h before and after). Suppression periods were defined by an EEG amplitude < 10 µV for ≥ 400 ms [6] and was calculated on 2 s epochs on the central derivation with the best signal (Fz-Cz or adjacent; BRAIN-QUICK, Micromed). We took the mean SR of the 2 h. The relation between concentration and duration of thiopental administration and the SR was analyzed with a linear mixed effect model with the R software (lme4 library). The diagnostic accuracy of thiopental concentration to predict a discontinuous EEG was analyzed with a receiver operating characteristic curve (ROC, pROC library). Data are presented as their median and interquartile range.

**Fig. 1:**
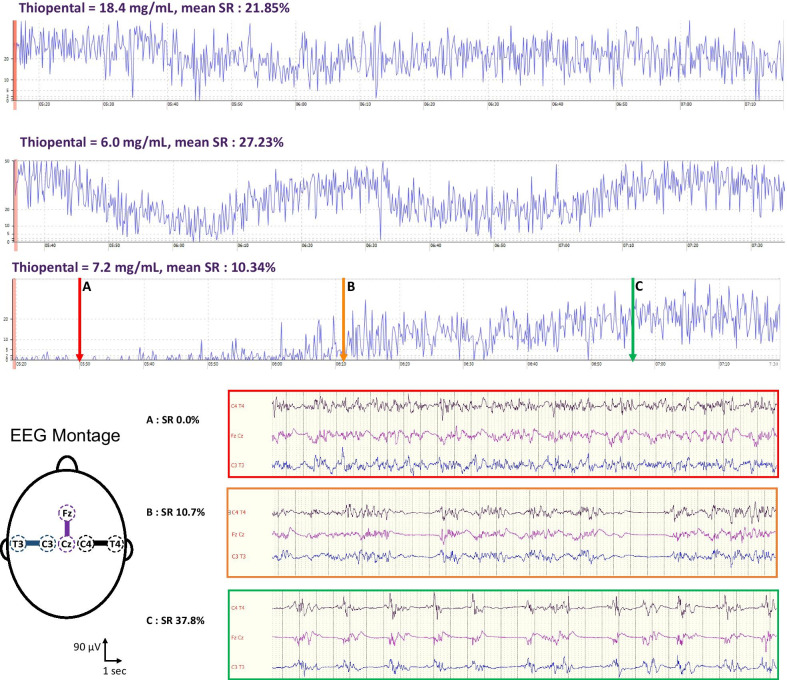
Examples of suppression ratio (SR) changes during the 2 h surrounding the blood sample time. Three different examples of SR changes are plotted in blue. The first two curves show similar SR in the same patient with a constant midazolam infusion, whereas thiopental concentrations vary by a ratio of 3. The last curve of another patient without midazolam, shows a different SR decay while the thiopental concentration is comparable to the one of the second curve. The lower panel presents three raw EEG samples of the lower SR curve (arrows A, B and C) using three bipolar derivations (right: C4–T4, midline: Fz–Cz, and left: C3–T3, see EEG montage of the left) with a 0.53 Hz high-pass filter and an 80 Hz low-pass filter

**Fig. 2:**
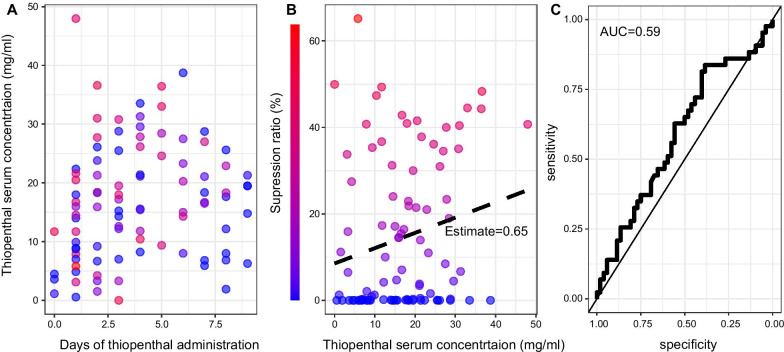
Suppression ratio and thiopental serum concentration relationship. **A** Thiopental serum concentration changes during the first ten administration days. The dot color represents the suppression ratio at the sampling time (the color scale is on the **B** panel). **B** Mean suppression ratio of the 2 h EEG time window around the blood sampling time of the thiopental serum concentration (*x*-axis). The dashed line represents the slope of the linear regression model (Estimate = 0.65 95%CI [0.09;1.21], *p* = 0.02). **C** Receiver operating characteristic curve of thiopental serum concentration to predict a SR ≥ 10% (AUC = 0.59 95% CI [0.47;0.71])

We included 30 patients, 47% (*n* = 14/30) had a refractory SE. They were 39.5 years old [27.5–55.5], 30% (*n* = 9) were female, and 37% had a potent thiopental adverse effect (sepsis: *n* = 5; hemodynamic instability: *n* = 6). The median concentration of thiopental (*n* = 95 samples) was 16.5 mg/ml [8.5–23.1], with a median administration rate of 2.1 mg/kg/h [1.3–2.9]. At the blood sampling time, 45.3% (*n* = 43/95) of the EEG were discontinuous with heterogeneous SR decay time (Fig. 1). 74% of the EEG from patients with a SE did not have epileptic discharges and 68% of patients with an IH had an ICP < 25 mmHg. The ICP value was not significantly associated with the thiopental concentration. There was a significant association between thiopental concentration and SR, which was not dependent on the indication (SE or IH). However, for a given thiopental concentration, the level of SR was highly variable and thiopental concentration could not predict a discontinuous EEG (AUC of the ROC = 0.59 95%CI [0.47; 0.71], Fig. 2). Other sedative agents were also administered in 20 patients but did not significantly influence SR; their withdraw led to a SR decrease in 4 cases.

The main limitation of the herein study is its retrospective design. Given the heterogeneity of the EEG changes for a given thiopental administration, we would argue to have a continuous SR monitoring when using barbiturate to reach a discontinuous activity. The concomitant monitoring of electrophysiological data and serum concentration might help tailor the individual administration and monitor the effect of thiopental on brain activity. Such strategy will need to be evaluated in a prospective trial.

## Data Availability

The datasets used during the current study are available from the corresponding author on reasonable request.
